# Quadriplegia from cervical osteomyelodiscitis with vertebral collapse: A case report

**DOI:** 10.1002/ccr3.6591

**Published:** 2022-11-15

**Authors:** Danay Herrera, Jose M. Acosta‐Rullan, Davide Fox, Livasky Concepcion, Jessica Hughes

**Affiliations:** ^1^ Internal Medicine HCA Florida Aventura Hospital Aventura Florida USA

**Keywords:** bacteria, infectious diseases, microbiology, MRSA, osteomyelitis, pathogen, Staph Aureus

## Abstract

Vertebral osteomyelitis is a rare clinical condition accounting for 1%–7% of all bone‐related infections. The increase in chronic diseases such as diabetes mellitus or those that lead to immunosuppression, the increase in spinal instrumentation including epidural catheters for pain management, and the continual increase in intravenous (IV) drug use are factors that have led to the rise in cases. The condition may present subtly without clinical signs and symptoms making early diagnosis difficult. Here, we present a rare case of spontaneous osteodiscitis of the cervical spine complicated by epidural abscess/phlegmon, burst fracture, and spinal cord injury due to methicillin‐resistant *Staphylococcus aureus* (MRSA) bacteremia in a patient with a history of intravenous drug use who presented with neck pain. The patient was treated with IV antibiotics and decompressive surgery and, however, was unable to regain the mobility of the lower extremities and regained only slight mobility in the upper extremities leading to an ultimate diagnosis of functional quadriplegia.

## 1 INTRODUCTION

Vertebral osteomyelitis makes up about 1 to 7% of all bone infections and is frequently seen in patients with a history of uncontrolled diabetes, chronic immunosuppression, or IV drug use.^1^, ^2^ Often, as stated by Acosta et al. (2004), it results from direct inoculation during spinal procedures, contiguous spread from soft tissue infections, and most commonly through hematogenous seeding.^1^ Giri et al. (2014) and Pfister H‐W et. Al (2014) report that *Staphylococcus aureus* remains the most common pathogen responsible for spinal infections followed by organisms such as *Mycobacterium tuberculosis*, *Brucella species*, *Aspergillus species*, *Candida species*, and *Cryptococcus neoformans*.^3^, ^4^ While the thoracic and lumbar spine are affected in a reported 35% and 50% of cases, the cervical spine accounts for only 3 to 10% of all cases, of which 27% have been reported in IV drug abusers.^1^ The diagnosis of cervical osteomyelitis, however, is often delayed because pain is usually attributed to degeneration in the elderly, and infection indicators such as fevers, elevated white blood cell counts, and C‐reactive protein levels may not always be present.^5^ Neurologic signs and symptoms such as weakness and numbness have been reported in a third of cases, and notably piercing pain may suggest that an epidural abscess is present.^6^ Because of the vague symptoms associated with vertebral osteomyelitis, a high index of suspicion, clinical judgment, and imaging studies are needed for an early diagnosis. Once diagnosed, the condition must be promptly treated with intravenous antibiotics and surgery, if required, for a more favorable prognosis.^3^ Here, we present a case of vertebral osteomyelitis/discitis due to MRSA bacteremia in a patient presenting with neck pain, bilateral paresthesia, and decreased motor strength with a history of IV drug use. In our case, the cervical spine was involved and osteodiscitis was complicated by extensive epidural abscess, bone osteodiscitis, and cord injury causing paresthesia and quadriplegia.

## 2 CASE PRESENTATION

**FIGURE 1:**
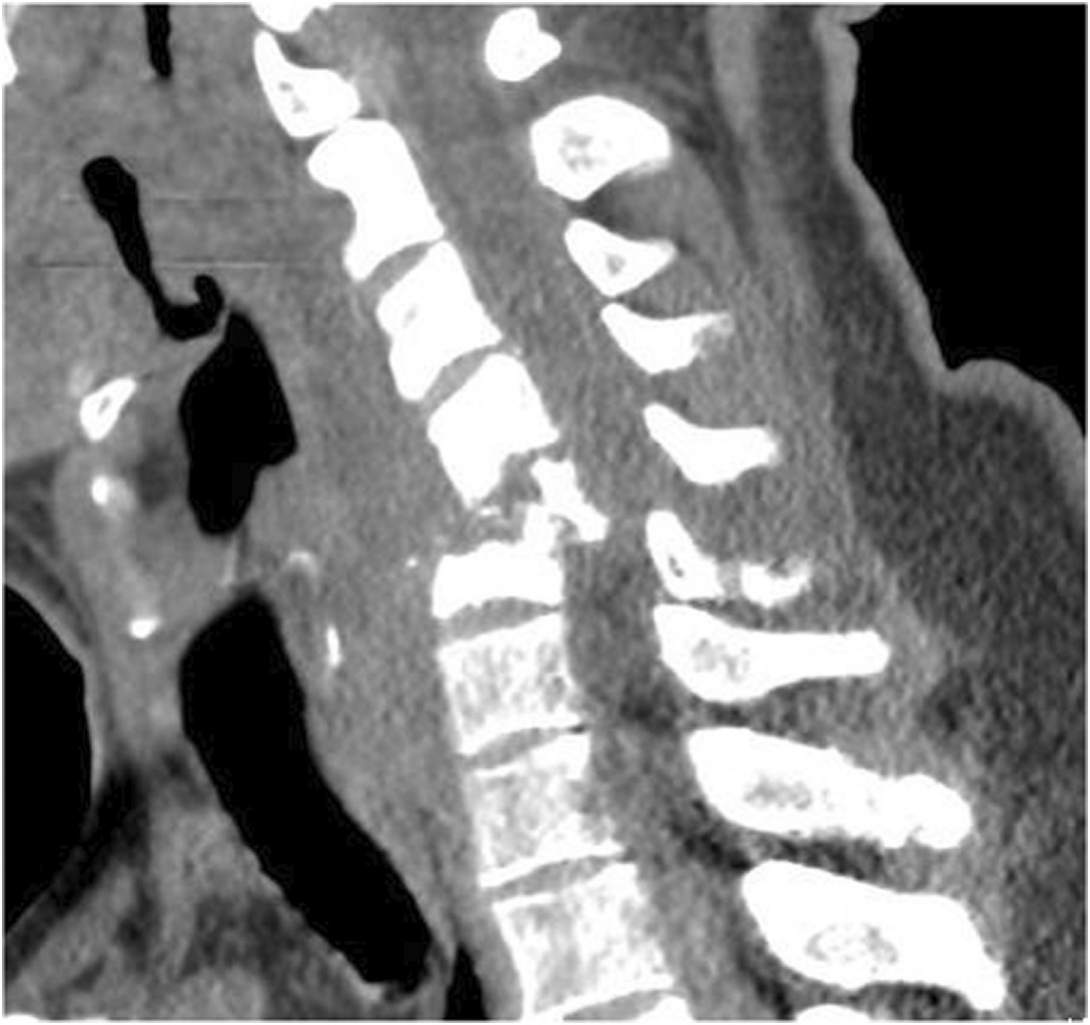
Sagittal CT view showing severe destructive changes at C4‐C5 resulting in severe canal stenosis due to osteomyelitis/diskitis.

A 40‐year‐old male with a past medical history of IV drug use and Hepatitis C presented to the hospital with a chief complaint of worsening neck pain and bilateral upper extremity paresthesia with associated motor deficits. The patient, a construction worker, reported the sudden onset of sharp neck pain while lifting a heavy box at work one week prior. He stated the pain had progressively worsened with radiation down the back and arms and was now associated with decreased hand grip strength and difficulty raising his arms above shoulder height. The patient admitted to IV fentanyl use for pain relief without reported improvement of symptoms. On presentation to the emergency department, his vital signs revealed a temperature of 98.7°F, blood pressure of 151/89 mmHg, heart rate of 62 bpm, and respiratory rate of 17 bpm. Upon initial physical examination, the patient appeared to be in distress demonstrating midline cervical spine tenderness greatest at C5 and C6, paraspinal cervical spine tenderness, and limited range of motion of the neck secondary to pain; however, no obvious bony deformity was noted on palpation of the cervical spine. The physical examination further revealed 4 out of 5 grip strength bilaterally, 4 out of 5 bilateral upper extremity strength with hyperreflexia, and numbness that extended from the neck down to the bilateral fingers. While a complete blood count did not reveal leukocytosis and a complete metabolic panel was within normal limits, sedimentation rate and c‐reactive protein were elevated on presentation at 58 mm/h and 4.1 mg/dl, respectively. Computerized tomography (CT) imaging in the emergency department demonstrated severe destructive changes at the C4‐C5 level disk space with the destruction of the C5 vertebral body suggesting burst fracture causing severe cervical spinal canal stenosis and cord injury (Figure 1). At the time, a cervical collar was placed for spine stabilization; however, the patient was noncompliant with strict neck movement precautions and was noted moving his neck while turning in bed. On re‐examination, the patient's physical examination showed worsening signs of neurological status with minimal voluntary movement of the upper extremities appreciated, 3 out of 5 bilateral upper extremity strength with markedly decreased ability to extend arms, and now 0 out of 5 bilateral grip strength, a significant difference from presentation prompting further imaging.

**FIGURE 2:**
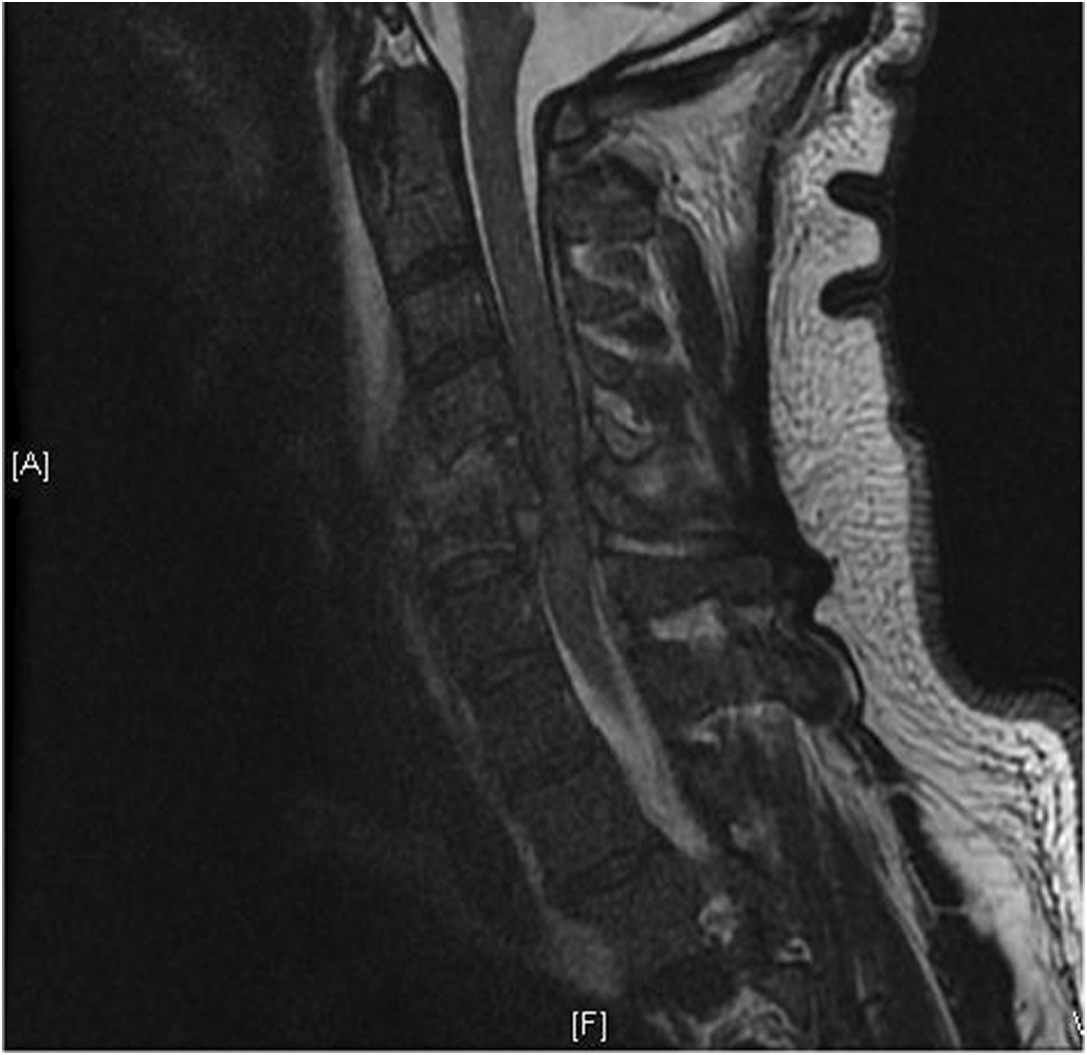
Sagittal MRI view showing diskitis/osteomyelitis at C4‐5 and C5‐6, with the collapse of the C5 vertebral body. Multilevel epidural phlegmon/abscess resulting in severe spinal stenosis and moderate cord compression from C4‐C6. Extensive prevertebral and paravertebral phlegmon/abscess is also seen.

**FIGURE 3:**
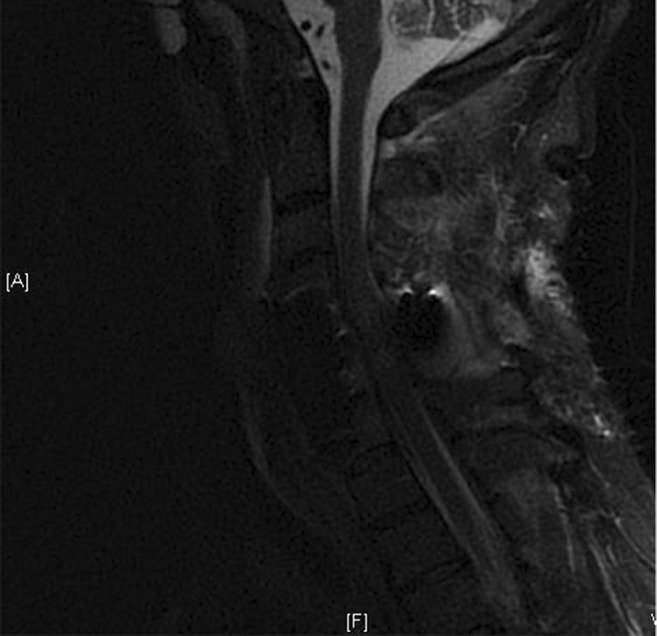
Sagittal MRI view status post‐anterior cervical discectomy and fusion and posterior spinal fusion hardware placement for epidural abscess, osteomyelitis, and diskitis with interval improvement of epidural abscess and cord compression.

Magnetic resonance imaging (MRI) demonstrated C3‐C4 broad‐based disk bulge, osteomyelitis/discitis in the C4‐C5 and C5‐C6 vertebral bodies with osseous destruction and bony retropulsion of C5 into the spinal cord, C5‐6 burst fracture, and kyphotic deformity, C4‐C5 severe cervical stenosis, and cord injury (Figure 2). Paravertebral muscular and soft tissue hyperintensity beginning at the C2 level, extending inferiorly to the C5 level consistent with extensive prevertebral and paravertebral phlegmon/abscesses was also noted. Two sets of blood cultures were obtained while in the emergency department, and the patient was then started on broad‐spectrum antibiotic therapy with Vancomycin 1500 mg every 6 hours, Cefepime 2 g every 8 hours, and Metronidazole 100 ml every 8 hours. Neurosurgery was called, and the patient was taken for emergent anterior decompression and fusion of C4 through C7 with the use of titanium plate and screws, complete corpectomies at C5 and C6, evacuation of epidural abscess, and arthrodesis anterior of kyphotic deformity. Intraoperative wound cultures were obtained at that time and sent to the microbiology laboratory for evaluation. The patient was returned to the operating room 4 days later for further cervical stabilization now with posterior C4 to C7 decompression fusion and open reduction and internal fixation of fractures C4, C5, and C6; however, improvement of neurological function was not achieved. Blood and wound cultures confirmed infection with Methicillin‐resistant *Staphylococcus aureus* (MRSA), and upon sensitivities, the patient was transitioned to Daptomycin 6 mg/kg IV to complete a 6‐week intravenous course of antibiotics per infectious disease recommendations. A follow‐up MRI of the cervical spine obtained 12 days after the initial MRI, Figure 3, showed adequate decompression of the spinal canal, but the patient did not regain bilateral lower extremity mobility and now has only minimal movement of upper extremities despite extensive physical and occupational therapy with an ultimate diagnosis of functional quadriplegia.

## 3 DISCUSSION

*Staphylococcus aureus* is the leading pathogen responsible for causing spinal epidural abscesses (SEA), accounting for about two‐thirds of reported cases. The most common sites for epidural abscesses are the thoracic spine, followed by the lumbar, and the least common is the cervical spine. Typically, at the time of diagnosis, there are several vertebral levels involved. The majority of abscesses are located at the posterior segments. The abscesses located anteriorly are typically associated with vertebral osteomyelitis.^6^ In our case, a SEA occurred in the cervical spine region located anteriorly and posteriorly from C4‐C6 with osseous destruction and deformity, which is an unusual presentation. Per Davis et al. (2004), a diagnostic triad of SEA consisting of fever, spinal pain, and neurologic deficits exist; however, rarely do patients have all three elements at the time of presentation.^7^ A study by Syuichi et al. (2019) demonstrated that 71% of the patients had back/neck pain, 66% had fevers, and 34% had paralysis.^8^

Spinal epidural abscesses (SEA) have four development stages: stage I: fever, back pain, and tenderness at the affected spine level; stage II: radicular pain (nerve root symptoms) radiating from the affected part of the spinal cord, nuchal rigidity/neck stiffness, and decreased tendon reflexes; stage III: neurological sensory deficits such as hypesthesia, paresthesia or dysesthesia, muscle weakness, bowel, or bladder dysfunction; stage IV: paralysis.^8^ Prompt intervention is mandatory if the progression of weakness or other neurologic findings are detected as paralysis is irreversible once it is evident. SEA at any level is a critical condition; it is particularly catastrophic in the upper cervical region due to the vulnerability of the atlantoaxial joint, as we can see in our case. Spinal cord compression at the cervical region can also impact breathing as the diaphragmatic innervation is from C3, C4, and C5.^9^ In our case, the patient presented in stage III with worsening neck pain and weakness in bilateral upper extremities with associated paresthesia, resulting in functional quadriplegia.

Surgical decompression was performed on the presented patient; however, his prolonged hospital course was complicated by the development of a mucus plug leading to the need for intubation and bronchoscopy, possibly as a result of phrenic nerve root involvement. According to Moustafa et al. (2019), decompression and removal of the abscess along with systemic antibiotic therapy is the cornerstone treatment.^10^ Immediate surgical intervention may be necessary if acute or progressive neurologic deficits are evident. Blood cultures should be obtained once the diagnosis is suspected along with empiric antibiotic coverage. For IV drug users, gram‐positive bacteria coverage empowers. The duration of antimicrobials is usually four to six weeks, varying from case to case depending on laboratory and imaging data. A follow‐up MRI should be obtained at four to six weeks if there is a significant improvement or if clinical deterioration occurs.^11^ Per Danner and Hartman (1987), reported outcomes include 54% complete recovery, 23% with residual weakness, 9% paralysis, and 14% death. Such is markedly influenced by the time from admission to proving the diagnosis, the location of the culprit lesion, and the severity of the neurological deficits before the initiation of treatment.^12^

## 4 CONCLUSION

Early diagnosis along with a high clinical index of suspicion and rapid intervention is crucial to ensure a better outcome in patients with SEA. A diagnosis of SEA should always be considered in patients presenting with progressive weakness and neurological deficits who have a history of IV drug use. MRI remains the preferred imaging modality to establish the diagnosis. Maximized awareness of the disease and a high clinical suspicion index are imperative for rapid intervention along with patient education to prevent worsening of clinical status. Education should be focused on bedrest, log‐roll precautions, and C‐collar compliance to avoid further neurological impairment or worsening of presenting deficit.

## AUTHOR CONTRIBUTIONS

The author Dr. Danay Herrera Hernandez completed the introduction, helped with the editing and re‐wording of the case presentation, gathered images from the radiology department, and edited the discussion. She further organized the document and completed the revision of the initial submission with the use of the comments from the reviewers. Dr. Jose M Acosta‐Rullan completed the discussion and conclusion sections and finalized edits of said sections. Dr. Davide Fox wrote the case presentation and obtained consent from the patient. Dr. Livasky Concepcion Perez helped with editing and revising the initial case write‐up. He first brought the case to the attention of the authors and helped with the planning for the submission. Dr. Jessica Hughes helped with editing and revising the initial case write‐up.

## FUNDING INFORMATION

The authors received no financial support for this article's research, authorship, and/or publication.

## CONFLICT OF INTEREST

The authors have no conflicts of interest to declare. All co‐authors have seen and agree with the contents of the manuscript, and there is no financial interest to report. We certify that the submission is original work and is not under review at any other publication.

## CONSENT

Written informed consent was obtained from the patient to publish this report in accordance with the journal's patient consent policy.

## DISCLAIMER

This research was supported (in whole or in part) by HCA Healthcare and/or an HCA Healthcare‐affiliated entity. The views expressed in this publication represent those of the author(s) and do not necessarily represent the official views of HCA Healthcare or any of its affiliated entities.

## Data Availability

Data sharing not applicable to this article as no datasets were generated or analyzed during the current study
